# Atrial Secondary Mitral Regurgitation Outcomes Following Mitral Transcatheter Edge-to-Edge Repair: Results From the EXPANDed Studies

**DOI:** 10.1161/CIRCINTERVENTIONS.125.015883

**Published:** 2026-01-21

**Authors:** Mark J. Ricciardi, Gagan Singh, Jason H. Rogers, Tobias Ruf, Wolfgang Rottbauer, Patrick Horn, Paul Mahoney, Bassem Chehab, Federico M. Asch, Jose Zamorano, Matthew J. Price, M. Andrew M. Morse, Michael J. Rinaldi, Paolo Denti, Melody Dong, Rong Huang, Francesco Maisano, Ralph Stephan von Bardeleben, Evelio Rodriguez, Saibal Kar

**Affiliations:** Endeavor Health Northshore Cardiovascular Institute, Evanston, IL (M.J. Ricciardi).; University of Chicago Pritzker School of Medicine, Chicago, IL (M.J. Ricciardi).; University of California-Davis, Sacramento (G.S., J.H.R., R.S.v.B.).; University Medical Center of the Johannes Gutenberg-University Mainz, Germany (T.R.).; Ulm University Heart Center, Germany (W.R.).; University Hospital Dusseldorf, Germany (P.H.).; University of Pittsburgh Medical Center Heart and Vascular Institute, PA (P.M.).; Ascension Via Christi, Wichita, KS (B.C.).; Cardiovascular Core Laboratories, MedStar Health Research Institute, WA (F.M.A.).; Ramón y Cajal University Hospital, Spain (J.Z.).; Scripps Clinic, CA (M.J.P.).; Ascension Saint Thomas, Nashville, TN (M.A.M.M., E.R.).; SangerHeart and Vascular Institute of Cardiothoracic Surgery, Charlotte, NC (M.J. Rinaldi).; San Raffaele University Hospital, Milan, Italy (P.D.).; Abbott Structural Heart, Santa Clara, CA (M.D., R.H.).; IRCCS Ospedale San Raffaele, Milan, Italy (F.M.).; Los Robles Regional, Thousand Oaks, CA (S.K.).

**Keywords:** atrial fibrillation, dilatation, echocardiography, prognosis, quality of life

## Abstract

**BACKGROUND:**

Atrial secondary mitral regurgitation (aSMR) is a distinct subtype of SMR characterized by normal leaflets, annular dilatation, left atrial (LA) enlargement, and preserved left ventricular function. Treatment pathways for aSMR are undefined, and limited data exist regarding outcomes following mitral transcatheter edge-to-edge repair (MTEER). The analysis aimed to evaluate outcomes in patients with aSMR treated with MTEER from the EXPANDed (Evaluation of the MitraClip X System Post-MArket Real-World CliNical Outcomes Database ) studies.

**METHODS:**

One-year outcomes were assessed in patients from the EXPANDed studies (EXPAND and EXPAND G4 [Evaluation of the MitraClip X System Post-MArket Real-World CliNical Outcomes Database Generation 4 ]) who met criteria for aSMR. aSMR was defined by the presence of atrial fibrillation, left ventricular ejection fraction ≥45%, and at least 1 dilated LA parameter per echocardiographic core laboratory assessment: LA volume index, LA diameter, or LA diameter index.

**RESULTS:**

Of the 967 patients with SMR treated with MTEER in the EXPANDed data set, 160 (17%) met criteria for aSMR. Patients with aSMR were elderly (78±8 years), symptomatic (Kansas City Cardiomyopathy Questionnaire Overall Summary score, 48±27 pts), and had small left ventricular and large LA dimensions at baseline. Acute procedural success was achieved in 97.5% of patients with aSMR, with MR reduction to ≤1+ in 95.2% at 1 year. There were significant 1-year improvements in quality of life (+19 pt Kansas City Cardiomyopathy Questionnaire Overall Summary) and functional capacity (New York Heart Association I/II 80%). The 1-year all-cause mortality rate was 9%, with patients experiencing a 56% reduction in 1-year heart failure hospitalization rates from pre- to post-MTEER.

**CONCLUSIONS:**

In the largest population of patients with aSMR assessed by an echocardiographic core laboratory, MTEER safely and significantly reduced MR with improvements in quality of life and reduction in heart failure hospitalization through 1 year.

**What is Known**

- Atrial secondary mitral regurgitation is a subgroup of secondary mitral regurgitation broadly defined by annular dilatation and left atrial enlargement, and preserved left ventricular function.
- Atrial secondary mitral regurgitation pathology, prognosis, and transcatheter edge-to-edge repair treatment strategies with contemporary devices are poorly defined.

**What the Study Adds**

- In the EXPANDed studies, patients with atrial secondary mitral regurgitation experienced significant mitral regurgitation reduction, substantial improvements in quality of life, reduction in heart failure hospitalization rate, and low rates of all-cause mortality through 1 year after transcatheter edge-to-edge repair.
- Treatment of atrial secondary mitral regurgitation with contemporary transcatheter edge-to-edge repair devices led to 97.5% acute procedural success and 95.2% mitral regurgitation reduction to ≤1+ at 1 year.

The heterogeneous nature of secondary mitral regurgitation (SMR) has become increasingly recognized in recent years as physicians seek optimal treatment pathways tailored to individual patients. Atrial SMR (aSMR) represents a distinct subtype of SMR characterized by mitral annular dilation and flattening and left atrial (LA) enlargement, and preserved left ventricular (LV) function.^1^ Patients with aSMR typically present with heart failure (HF) with preserved ejection fraction (HFpEF), for which the efficacy of standard HF therapy is less clear due to limited guideline recommendations for this subtype. Current HFpEF management is limited to diuretics, SGLT2 inhibitors, or noninterventional treatment, with no specific guidance on the treatment for aSMR.^2^

Although mitral transcatheter edge-to-edge repair (MTEER) has been investigated for ventricular SMR in trials such as COAPT (Cardiovascular Outcomes Assessment of the MitraClip Percutaneous Therapy Trial),^3^ MITRA-FR (Multicentre Study of Individualized Treatment by Repair of Abnormal Mitral Valve—FRanceMulticentre Study of Individualized Treatment by Repair of Abnormal Mitral Valve—FRance),^4^ and RESHAPE-HF2 (Randomized Evaluation of the Safety and Health Assessment of the Percutaneous Edge-to-Edge Repair of the Mitral Valve in Heart Failure Patients: 2nd Trial),^5^ the outcomes of MTEER in patients with aSMR remain less clear. An additional challenge in assessing aSMR is the lack of a standardized definition, with studies employing varying criteria based on LV and LA parameters, atrial fibrillation (AF), and HFpEF definitions used.^6–9^

Although patients with aSMR may be considered more difficult to treat with MTEER, evidence supporting or refuting this notion is limited.^10^ These latest generations of MitraClip MTEER devices safely and effectively treat patients with MR.^11–13^ Clipping strategies have also evolved to treat more complex anatomies and phenotypes with MTEER.^14–16^ This analysis aimed to evaluate 1-year outcomes of patients with aSMR treated with the third and fourth generations of the MitraClip System from the EXPANDed (Evaluation of the MitraClip X System Post-MArket Real-World CliNical Outcomes Database) data set.

## Methods

### Analysis Population

The EXPANDed data set includes EXPAND and EXPAND G4 (Evaluation of the MitraClip X System Post-MArket Real-World CliNical Outcomes Database Generation 4), with details previously published^11–13^ and present in Supplemental Methods. Both studies received regulatory approval from relevant institutional review boards or ethics committees and national competent authorities, as applicable per country requirements. These studies adhered to good clinical practice standards outlined in the Declaration of Helsinki; all patients provided written informed consent.

aSMR was defined as SMR with a history of AF, normal mitral valve leaflets, an LV ejection fraction ≥45%, and the presence of 1 or more of the following dilated LA: LA volume index >34 mL/m^2^, LA diameter (LAD) >4 cm (males) or >3.8 cm (females), and LAD index >2.3 cm/m^2^. This definition was chosen in alignment with the previously proposed aSMR definition and available echocardiographic data.^17^ LA volume and diameter thresholds were based on upper normal limits defined as 1 SD above the mean reported in the American Society of Echocardiography guidelines.^18^

Patients were excluded if they had a primary or mixed MR cause, nonevaluable MR cause, missing baseline echo data, or did not meet the aSMR criteria. Analysis on the MitraClip G4 System only included patients from EXPAND G4. A cutoff of 4.5 cm for LAD, based on the lower third quartile of LAD in the EXPANDed aSMR population, was used to define patients with large (>4.5 cm) or smaller LADs (≤4.5 cm). A cutoff of 3.3 cm for anterior-posterior diastolic annular dimension (APDAD) and 5 cm^²^ for mitral valve area (MVA), based on the median values in the EXPANDed aSMR population, was used to define patients with large or smaller APDADs and MVAs. Additional study definitions are provided in Supplemental Methods.

### Statistical Analysis

Statistical methods employed both descriptive and inferential analyses. Continuous variables were presented as mean±SD or median (interquartile range), as appropriate, and were compared using an ANOVA or the Kruskal-Wallis test for nonparametric data. Student *t* test was used for paired continuous data. LV echo parameters and Kansas City Cardiomyopathy Questionnaire Overall Summary were analyzed for patients with complete baseline, 30-day, and 1-year data. Categorical variables were presented as percentages of available data and were compared using the Fisher exact or χ^2^ test. The Bowker test was used for paired nominal data. Major adverse events and device-related complications 1 year after the index procedure were reported in patients who had adverse events or did not withdraw from the study before the lower limit of the visit window. The Kaplan-Meier method was used to estimate all-cause mortality and heart-failure hospitalization (HFH) at 1 year with log-rank tests for group comparisons; patients were censored at their last known event-free date. A 2-sided *P* value of <0.05 was considered statistically significant. Statistical analyses were performed using SAS version 9.4 (SAS Institute Inc, Cary, NC).

## Results

### Analysis Population

**Figure 1.**
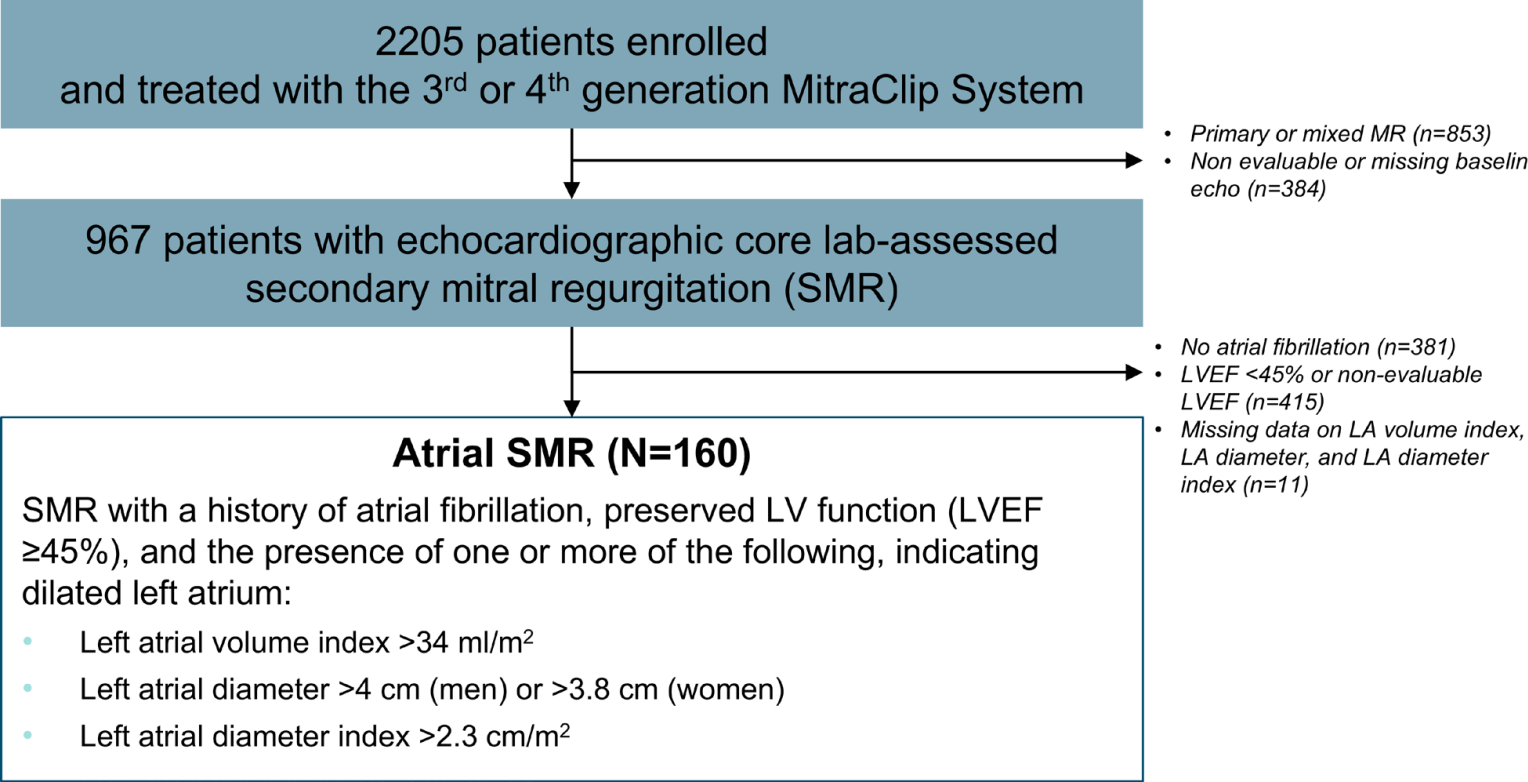
**Analysis population.** LA indicates left atrial; LV, left ventricle; LVEF, left ventricular ejection fraction; MR, mitral regurgitation; and SMR, secondary mitral regurgitation.

A total of 2205 patients were included in the EXPANDed data set, of whom 967 patients were classified with SMR. Of these, 160 patients met the criteria for aSMR per analysis definition (Figure 1). Half of these patients were women, and the mean age was 78 years (Table 1). Compared with the overall SMR population from EXPAND and EXPAND G4,^11–13^ mean LV ejection fraction was preserved (56.3%), and LV dimensions were smaller. LA volumes were larger (120 mL for aSMR).

**Table 1.**
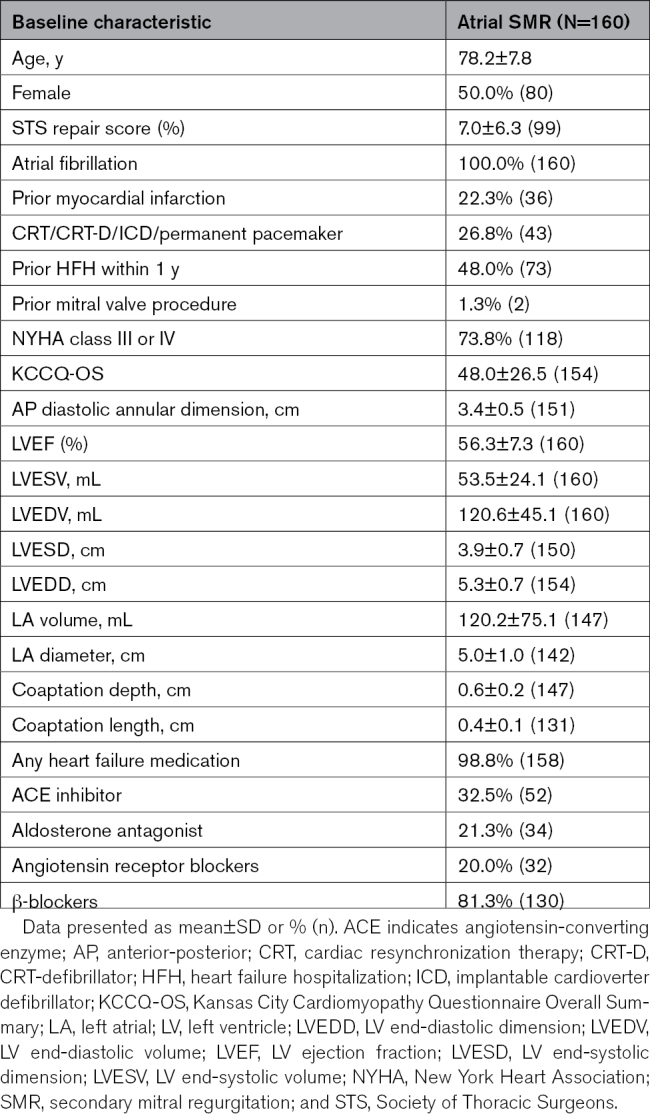
Baseline Characteristics

### Procedural Outcomes

**Figure 2.**
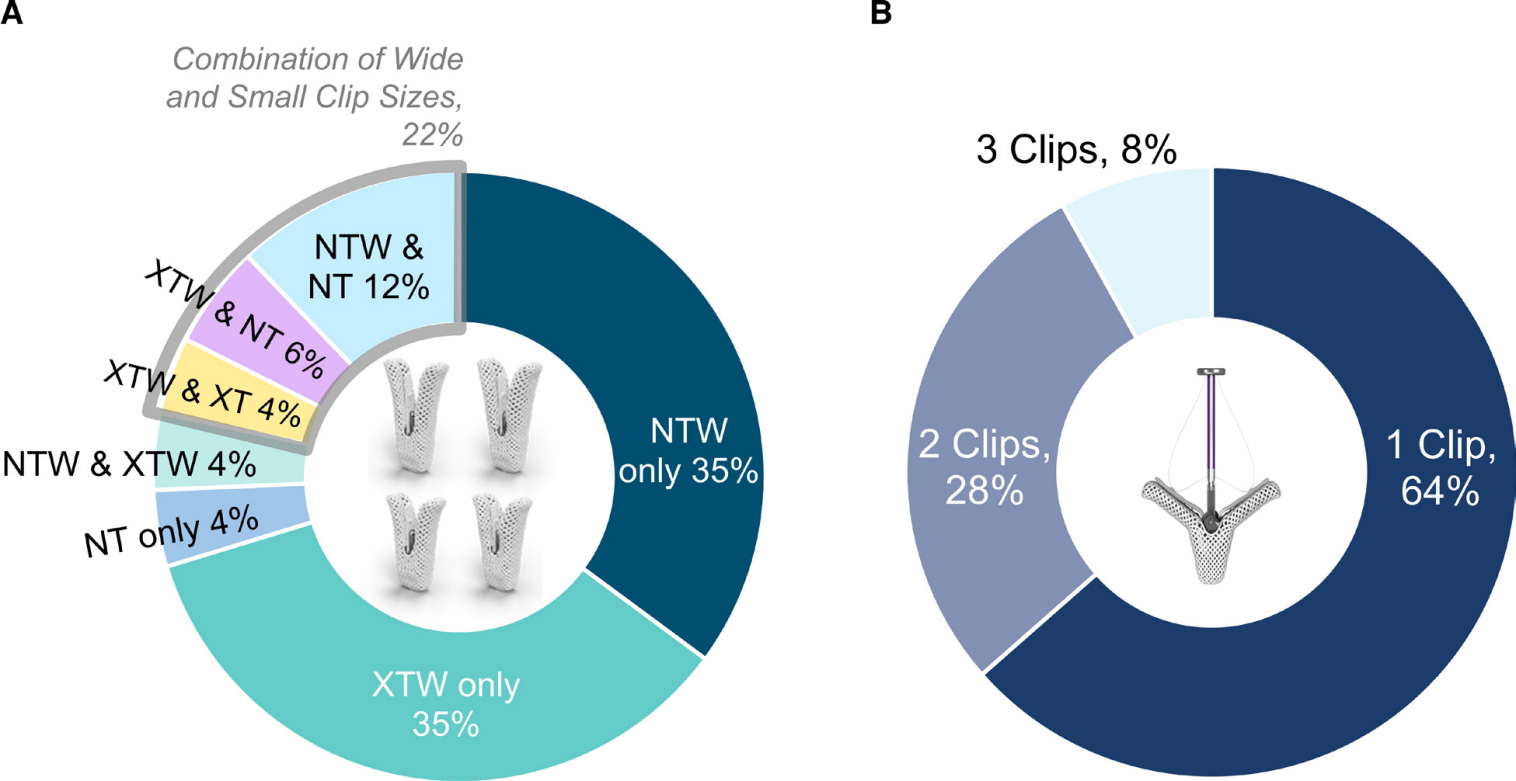
**Clip usage with the MitraClip G4 System.** A variety of clip combinations **(A**) were with most patients treated with 1 clip (**B**).

Acute procedural success was achieved in 97.5% of patients (Table 2)—the 4 patients who did not achieve acute procedural success were from the EXPAND study: 1 did not have a clip implanted, 1 died before discharge, and 2 had residual 3+ MR at discharge or 30 days. An average of 1.4 clips were implanted per patient with a median device time of 45 minutes. The mean mitral valve gradient increased slightly post-MTEER from 1.7±0.8 mm Hg at baseline to 3.8±1.8 mm Hg at 30 days (64 patients, paired analysis), with no clinically significant change indicating stenosis. There were no instances of SLDA, embolization, or leaflet damage through 30 days. An SLDA was noted in 1 patient after 30 days, and the patient underwent a mitral valve replacement surgery. Procedural outcomes were more favorable with the MitraClip G4 system, with 100% acute procedural success. The most common clip combinations were XTW or NTW alone (35% each; Figure 2).

**Table 2.**
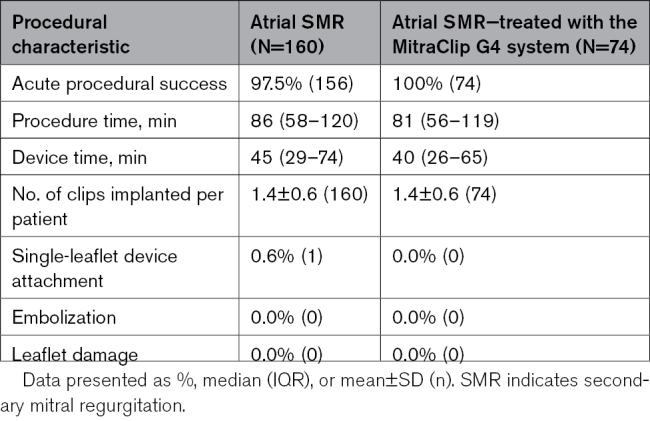
Procedural Characteristics

In patients with smaller LADs (≤4.5 cm), 1 clip was used in most cases (89%), and the remaining with 2 clips (11%; Figure S1). In contrast, 50% of patients with larger LADs (>4.5 cm) were treated with 1 clip, followed by 38% with 2 clips and 12% with 3 clips. Most patients with smaller LADs were treated with XTW alone (42%) or NTW alone (42%). In patients with larger LADs, an XTW clip was used in 52% of patients. Similar outcomes were observed by baseline APDAD and MVA (Figures S2 and S3). Most patients received clips on A2P2 segments regardless of baseline APDAD, LAD, or MVA (Table S1).

### Echocardiographic Outcomes

**Figure 3.**
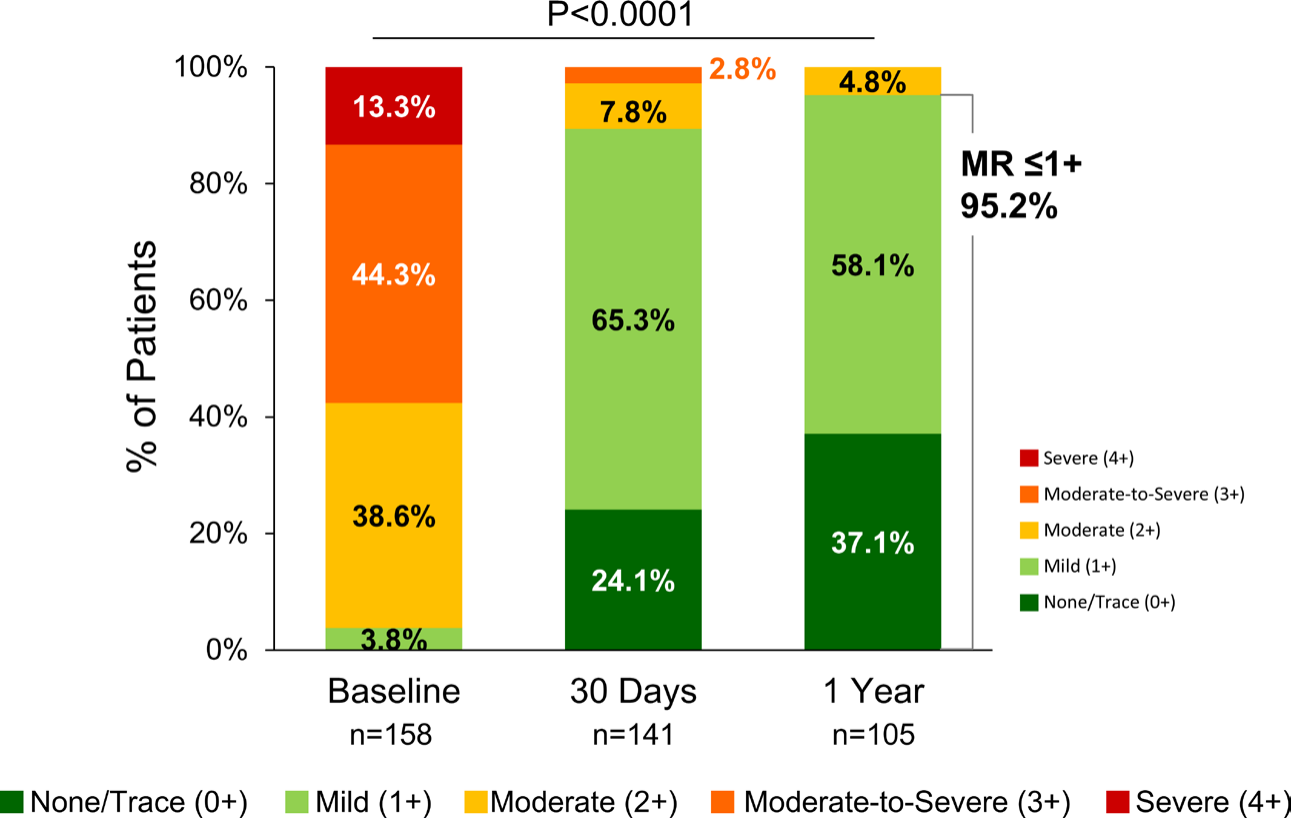
**Mitral regurgitation (MR) through 1 year.** Severity based on independent echocardiographic core laboratory assessment. Unpaired data shown; significance by χ^2^ test (paired comparison, baseline and 1 year, n=99).

There was significant MR reduction at 30 days and 1 year compared with baseline (*P*<0.001; Figure 3). In the EXPAND G4 cohort, significant MR reduction was observed at 1 year (*P*<0.001) with 98% of patients achieving MR ≤1+ at 1 year. Of these patients, 100% of patients with aSMR with smaller LADs achieved MR ≤1+, and 97% of patients with aSMR with larger LADs achieved MR ≤1+; 97% of patients with aSMR with smaller APDADs and MVAs also achieved MR ≤1+, and 95% of patients with aSMR with larger APDADs or MVAs achieved MR ≤1+ at 1 year. LV end-diastolic volume significantly decreased from baseline (123 mL) to 30 days (113 mL; *P*=0.002) and to 1 year (106 mL; *P*<0.001). No significant changes in LV end-systolic volume were observed (Figure S4).

### Clinical and Safety Outcomes

**Figure 4.**
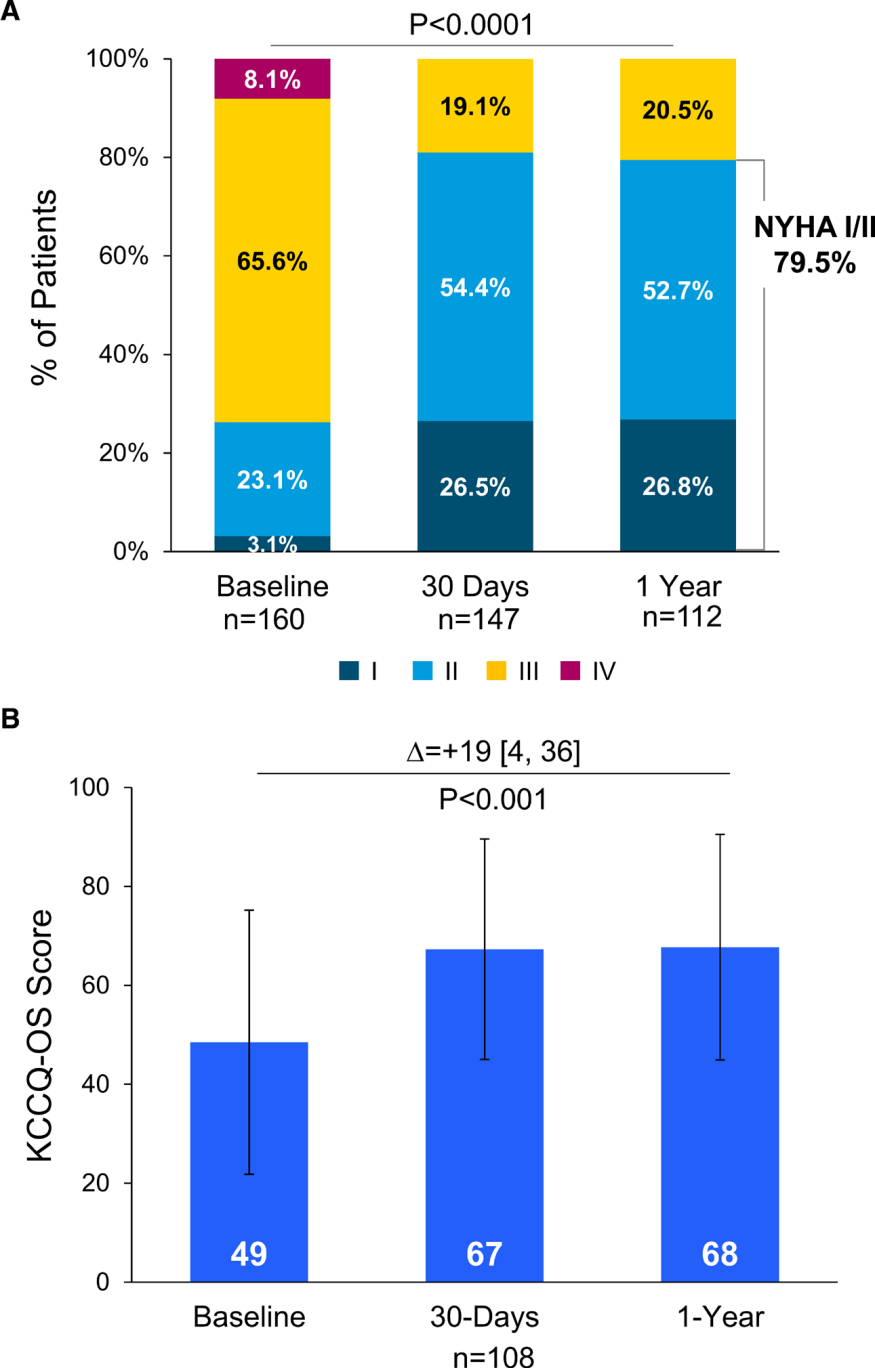
**Functional capacity and quality of life for patients through 1 year.** A significant increase in both New York Heart Association (NYHA) functional class (**A**) and Kansas City Cardiomyopathy Questionnaire Overall Summary (KCCQ-OS) score (**B**) was observed. For KCCQ-OS, data are presented as mean±SD.

**Figure 5.**
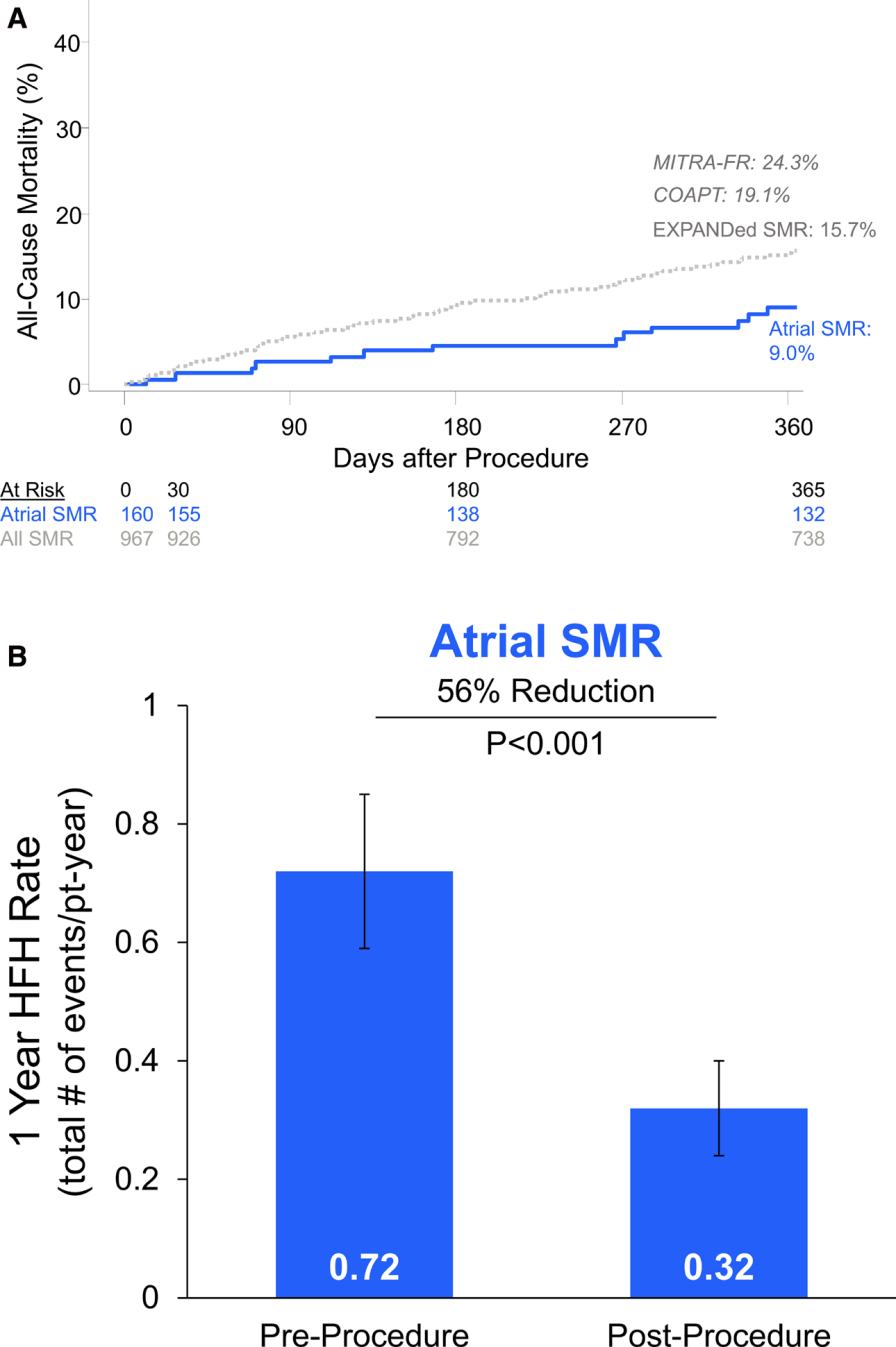
**Kaplan-Meier all-cause mortality through 1 year and change in annualized heart failure hospitalization (HFH) rate.** One-year rate of all-cause mortality was lower for patients with atrial secondary mitral regurgitation (SMR) compared with the entire EXPANDed (Evaluation of the MitraClip X System Post-MArket Real-World CliNical Outcomes Database) SMR cohort, COAPT trial (Cardiovascular Outcomes Assessment of the MitraClip Percutaneous Therapy Trial) ^3^ or MITRA-FR (Multicentre Study of Individualized Treatment by Repair of Abnormal Mitral Valve—FRance) trial^4^ (**top**). Annualized HFH decreased with atrial SMR compared with the year before the index procedure. Data presented as rates with error bars indicating the 95% CI (**bottom**).

At 1 year, a significant increase in patients with aSMR in New York Heart Association functional class I/II was observed (79.5% at 1 year versus 26.2% at baseline, *P*<0.0001; Figure 4). Patients with aSMR experienced a significant increase in Kansas City Cardiomyopathy Questionnaire Overall Summary score at 1-year follow-up (∆+19 [4–36], *P*<0.001). There was a slight decrease in the use of ACE (angiotensin-converting enzyme) inhibitors and β-blockers, but a slight increase in antiarrhythmic medications (Table S2). Kaplan-Meier 1-year mortality rate was 9.0% for patients with aSMR; this rate was 15.7% for all patients in EXPANDed with SMR (Figure 5). Annualized HFH rates decreased after MTEER, with a 56% reduction in the annualized HFH rate observed (Figure 5). Through 1 year, there were 3 instances of myocardial infarction, 1 stroke, and 4 mitral valve replacements (Table 3); 15 (9.4%) of patients with aSMR experienced 24 adverse events related to a cardiac arrhythmia, with 5 occurring within 30 days. Thirteen (13) patients received a rhythm control treatment, including antiarrhythmic medications (8), cardioversion (4), ablation (3), pacemaker (3), and LA appendage occlusion (2). There was 1 SLDA reported after the 30-day follow-up (mentioned previously) and no instances of device embolization in these patients.

**Table 3.**
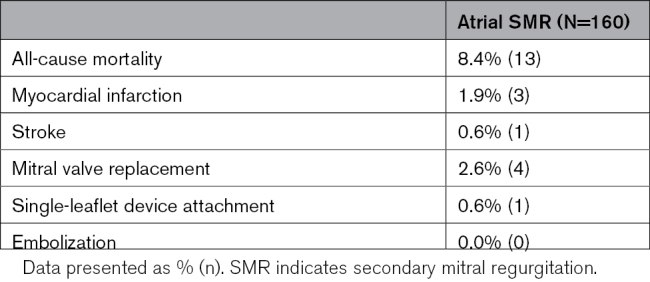
Major Adverse Events Through 1 Year

## Discussion

In this analysis of the EXPANDed data set, patients with aSMR achieved significant MR reduction and improvements in functional status and quality of life through 1 year following MTEER regardless of clipping strategy. One-year rates of annualized HFH and mortality were comparable or lower relative to all patients with SMR in EXPANDed, and mortality rates were lower for patients with aSMR in EXPANDed than those reported in the COAPT^3^ and MITRA-FR^4^ trials. Among patients with larger LADs, multiple clips were more often used, often combining a wide clip and a standard clip.

A major challenge for aSMR is in the range of definitions for this phenotype. Although a definition was presented by an expert panel viewpoint in Zoghbi et al,^7^ other thresholds have been previously used. In addition to the criteria used in this analysis, aSMR has also been defined by the following: absence of wall abnormalities,^6,9,17,19^ annular dilation,^7^ differing cutoffs for LV ejection fraction (≥40% to ≥50%),^7,8,17^ normal LV size,^7,9,10,20–23^ flattened leaflets or coaptation line,^24^ and prior concomitant procedures or valvular disease.^8,25–27^ The variety of definitions complicates synthesizing existing data of aSMR and MTEER outcomes. Consequently, reported outcomes have varied, and many studies on aSMR have been single-center studies^8,25,26^ with limited patient and unstandardized measurements of echo parameters.^9,19,21^ With broader adoption of standardized imaging protocols, the refinement of aSMR should be collected in subsequent studies.

This lack of consensus is further reflected in the absence of dedicated clinical guidelines for aSMR.^28–30^ The 2025 European Society of Cardiology / European Association for Cardio-Thoracic Surgery valvular guidelines provided a class 2b recommendation for aSMR and a class 1 recommendation for ventricular SMR.^31^ The American College of Cardiology/the American Heart Association valvular guidelines provide a Class 2b recommendation for mitral valve surgery in severe SMR, atrial annular dilation, and preserved LV systolic function despite medical therapy, including AF therapy.^32^ Similarly, HF guidelines also have a Class 2b recommendation for mitral valve surgery in patients with severe SMR with HFpEF (LV ejection fraction ≥50%) despite medical therapy, but do not distinguish between ventricular and aSMR.^28^

Current practices suggest SGLT2 inhibitors and diuretics may facilitate reduced LA pressure in patients with HFpEF. With AF common in patients with HFpEF and aSMR’s etiologic relationship to AF, sinus rhythm restoration with cardioversion or ablation may improve diastolic function and LA function. Prior studies suggest that restoration of sinus rhythm in patients with early SMR who begin to show mild to moderate MR and LA enlargement may be useful in preventing progression to a full aSMR phenotype.^33^ In this analysis of EXPANDed, a slight increase in antiarrhythmic medication use was observed after MTEER, while the use of other HF medications decreased, which may reflect real-world tolerance and regimen of guideline-directed medical therapy. Moreover, only a quarter of patients with aSMR were treated with rhythm control strategies at baseline, and few experienced cardiac arrhythmia events following MTEER, suggesting patients were stable regarding their arrhythmias before and after MTEER.

MTEER has evolved since early trials.^3,4,34,35^ Previous outcomes from EXPAND G4 have demonstrated that MTEER is suitable in anatomies previously considered unclippable, with high procedural success and significant MR reduction to ≤1+.^14^ Fewer TEER devices per procedure are being used with shorter procedural times.^11^ In our present analysis, most patients with aSMR were treated with 1 clip, and preference was apparent for wide clips (NTW, XTW). In patients with LADs larger than 4.5 cm, 50% of patients were treated with ≥2 clips in contrast to only 11% of patients with smaller LADs ≤4.5 cm. A combination of wide and small clip sizes was used in 26% of these patients, while only 5% of patients with smaller LADs were treated with combinations. These results illustrate 2 important findings in current treatment options for aSMR: (1) available clip combinations effectively reduce MR with minimal-to-no safety events, including SLDA, in patients with aSMR, and (2) the existing toolbox clips were applicable in patients with more dilated atria.

### Limitations

Sample sizes of the analysis population were constrained by the availability of data that met the study’s definition, and follow-up was limited to 1 year. LA follow-up measurements were particularly limited in availability in the EXPANDed data set, and regional wall motion abnormalities and systolic mitral annular dimensions were not collected. Ventricular SMR could not be exclusively distinguished from aSMR, as not all of the criteria and thresholds were available or evaluable for all patients in EXPANDed. Patients included in the EXPANDed data set were deemed anatomically suitable for MTEER and may not represent the broader spectrum of patients with aSMR seen in routine clinical practice. Sites in EXPAND and EXPAND G4 were highly experienced with the MitraClip device. Additionally, there is no widely adopted clinical definition of aSMR, and therefore, study outcomes may not be translatable to other differently defined aSMR populations. Finally, medication therapy data were limited to baseline and 30 days only in EXPANDed, with no dosage data collected. Newer medications, such as sacubitril/valsartan and SLGT2 inhibitors, were unavailable or not widely used at the time of EXPAND or EXPAND G4.

### Conclusions

The prevalence of aSMR is expected to rise with increasing rates of AF and HFpEF,^36^ thereby highlighting the need to understand treatment options for aSMR. In this analysis of the EXPANDed data set—the largest population of patients with aSMR assessed by an ECL—patients with aSMR experienced improvements in functional capacity, quality of life, and achieved durable MR reduction through 1 year. Low rates of all-cause mortality and annualized HFH were observed, and rates were comparable or lower to those seen previously. These findings support the continued evaluation of MTEER in aSMR.

## Article Information

### Acknowledgments

The authors thank all sites and study principal investigators who contributed to the EXPAND and EXPAND G4 studies. We also thank Meridith Doyle, PhD, for assistance with writing.

### Sources of Funding

EXPAND (NCT03502811) and EXPAND G4 (Evaluation of the MitraClip X System Post-MArket Real-World CliNical Outcomes Database Generation, NCT04177394) studies were sponsored by Abbott.

### Disclosures

Dr Ricciardi has received consulting, speaker, and proctor fees and institutional grants from Abbott, Edwards, and Medtronic. Dr Singh has received consulting fees and honoraria from Abbott, Boston Scientific, InnovHeart, Medtronic, Philips Medical, W.L. Gore & Associates, and Shockwave Medical. Dr Rottbauer has received consulting fees/speaker honoraria from Abbott, Bayer Healthcare, Boston Scientific, Daiichi Sankyo, Edwards Lifesciences, and Medtronic; and is a member of the steering committee of the EXPAND G4 study (Evaluation of the MitraClip X System Post-MArket Real-World CliNical Outcomes Database Generation 4) for Abbott and Encourage AF study for Daiichi Sankyo. Dr Mahoney is a consultant and proctor for Medtronic, Edwards Lifesciences, and Boston Scientific; is a consultant for Abbott; and has been awarded research grants from Edwards Lifesciences, Medtronic, Abbott, and Boston Scientific. Dr Chehab has received study grants and consulting fees from Abbott, Edwards Lifesciences, and BioTronics. Dr Asch’s work as an academic core laboratory director is performed through institutional research grants (MedStar Health) with Abbott, Boston Scientific, Medtronic, Edwards Lifesciences, Neovasc, Ancora Heart, LivaNova, MVRx, InnovHeart, Polares Medical, and Aria Cardiovascular. Dr Zamorano has received speaker honoraria from Pfizer, Amgen, and Daiichi Sankyo; and research grants from Abbott and Edwards Lifesciences. Dr Price has received consulting fees and honoraria from Abbott, Boston Scientific, InnovHeart, Medtronic, Philips Medical, W.L. Gore & Associates, and Shockwave Medical. Dr Morse is a consultant for Edwards Lifesciences. Dr Rogers is a consultant to Abbott Structural Heart, Biosense Webster, and Boston Scientific. Dr Denti has received speaker honoraria from Abbott and Edwards Lifesciences; and has been a consultant to InnovHeart, Artiness, and Pi-Cardia. Dr Rinaldi has been awarded honoraria and consulting fees from Abbott, Boston Scientific, and Edwards Lifesciences. Dr Dong and R. Huang are employees of Abbott Structural Heart. Dr Maisano has received grants and institutional research support from Abbott, Medtronic, Edwards Lifesciences, Biotronik, Boston Scientific, New Valve Technology, and Terumo; has received honoraria and consulting fees (personal and institutional) from Abbott, Medtronic, Edwards Lifesciences, Xeltis, and Cardiovalve; has received royalty income and intellectual property rights from Edwards Lifesciences; and is a shareholder (including stock options) of CardioGard, Magenta, SwissVortex, Transseptal Solutions, Occlufit, 4Tech, and Perifect. Dr von Bardeleben has performed nonpaid trial activities for Abbott, Edwards Lifesciences, Medtronic, and the University of Göttingen (IIT) and serves as an advisory board or speaker bureau member for Abbott, Edwards Lifesciences, Medtronic, and NeoChord. Dr Kar has received grants and institutional research support from Abbott, Boston Scientific, and Edwards Lifesciences; consulting fees/honoraria from Abbott, Boston Scientific, W.L. Gore, and Medtronic; and served as national principal investigator of EXPAND and REPAIR MR (Abbott). Dr Rodriguez has been awarded grants and support for research from Abbott, Edwards Lifesciences, Boston Scientific, AtriCure, and CardioMech; and honoraria or consulting fees from Abbott, Edwards Lifesciences, Philips, Teleflex, and CardioMech. The other authors report no conflicts.

### Supplemental Material

Supplemental Methods

Tables S1–S2

Figures S1–S4

## Supplementary Material


