# Supplementary material for: Atrial Secondary Mitral Regurgitation Outcomes Following Mitral Transcatheter Edge-to-Edge Repair: Results From the EXPANDed Studies
Source: Circ Cardiovasc Interv. 2026 Jan 21;19(2):e015883. doi: 10.1161/CIRCINTERVENTIONS.125.015883 (PMC12915549; doi:10.1161/CIRCINTERVENTIONS.125.015883)
Supplement: Supplementary file 1 [file hcv-19-e015883-s001.pdf]

## Supplemental Materials


| | Page(s) |
|---|---|
| **Supplemental Methods** | 2-3 |
| **Supplemental Figure S1.** Clip Usage with the MitraClip G4 System in Atrial Secondary Mitral Regurgitation Patients by Left Atrial Diameter. | 4 |
| **Supplemental Figure S2.** Clip Usage with the MitraClip G4 System in Atrial Secondary Mitral Regurgitation Patients by Anterior Posterior Diastolic Annular Dimension. | 5 |
| **Supplemental Figure S3.** Clip Usage with the MitraClip G4 System in Atrial Secondary Mitral Regurgitation Patients by Mitral Valve Area. | 6 |
| **Supplemental Figure S4.** Left Ventricular (LV) Reverse Remodeling Through One Year. | 7-8 |
| **Supplemental Table S1.** Clip Location by Baseline Anatomic Characteristic. | 9 |
| **Supplemental Table S2.** Medication Usage Through 30 Days. | 10 |

## SUPPLEMENTAL METHODS

*Study Design*

The EXPANDed dataset represents a pooled, patient-level cohort of the EXPAND and EXPAND G4 studies, including 2205 patients with symptomatic moderate-to-severe and severe degenerative or secondary MR. The EXPAND and EXPAND G4 studies are global, prospective, multicenter, contemporary studies designed to evaluate the safety and effectiveness of the 3rd and 4th-generation MitraClip Systems, respectively. Patients were enrolled across 91 centers in the United States, Europe, Canada, Israel, Saudi Arabia, and Japan from 2018 to 2022. All patients met eligibility criteria based on the study protocols and local recommendations. The studies are registered at ClinicalTrials.gov (EXPAND, NCT03502811 and EXPAND G4, NCT04177394), and additional study details have been previously published.[12-14] Data, methods, and materials used to conduct the research will not be made available without permission.

*Study Definitions*

Patients were followed up at discharge, 30 days, and 1-year post-procedure. Transthoracic and transesophageal echocardiograms were performed at baseline; TTEs were performed at follow-up timepoints. All echocardiograms were retrospectively evaluated by independent echocardiographic core laboratories. Medical Research Development S.L. (Madrid, Spain) and MedStar Health Research Institute (Washington DC, USA) evaluated mitral valve anatomic characteristics and LV parameters for the EXPAND and EXPAND G4 cohorts, respectively. MedStar Health Research Institute assessed MR severity, etiology, and LA parameters of all EXPANDed patients following the ASE guidelines.[20,21] LA dimension was assessed using the parasternal long axis view. MR severity assessments were performed using a previously published multiparametric algorithm adapted from the criteria recommended by ASE guidelines.[22]

Clinical outcomes included functional status assessed by the New York Heart Association (NYHA) functional class, quality of life assessed by the Kansas City Cardiomyopathy Questionnaire-23 Overall Summary (KCCQ-OS) score, all-cause mortality, and HF hospitalizations (HFH) through 1 year.

Acute procedural success (APS) was defined as achieving a residual MR grade of 2+ or less at the time of discharge without death or mitral valve replacement surgery. Major adverse events were defined as death, myocardial infarction, stroke, mitral valve replacement, single leaflet device attachment (SLDA), and device embolization. Major adverse events were site reported through 1 year and adjudicated by an independent clinical evaluations committee through 30 days in EXPAND. Device-related complications such as SLDA, device-related embolization, and the need for surgical mitral valve replacement through 1-year were assessed and adjudicated by the echocardiographic core laboratory.

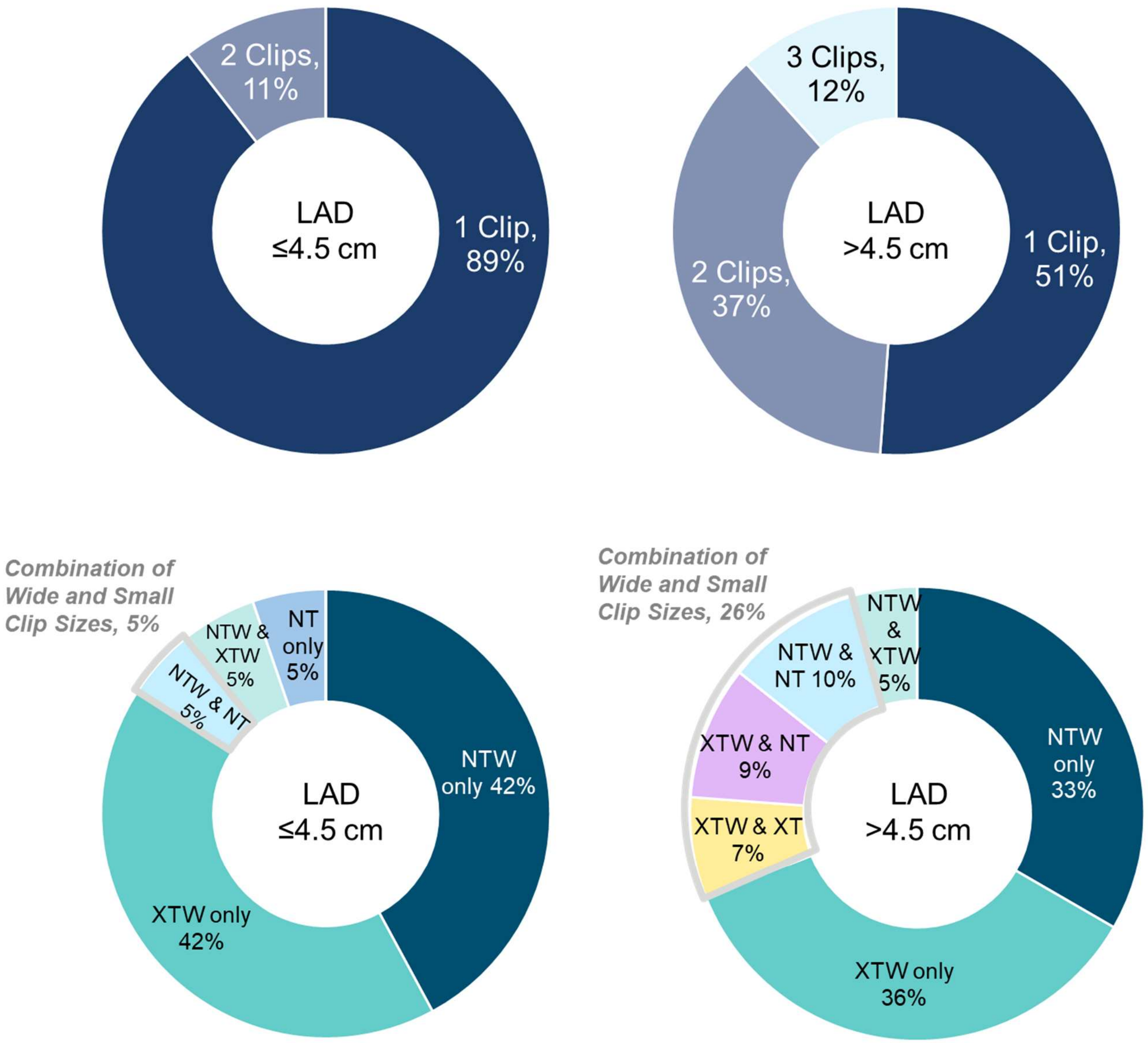


**Supplemental Figure S1. Clip Usage with the MitraClip G4 System in Atrial Secondary Mitral Regurgitation Patients by Left Atrial Diameter.** LAD, left atrial diameter.

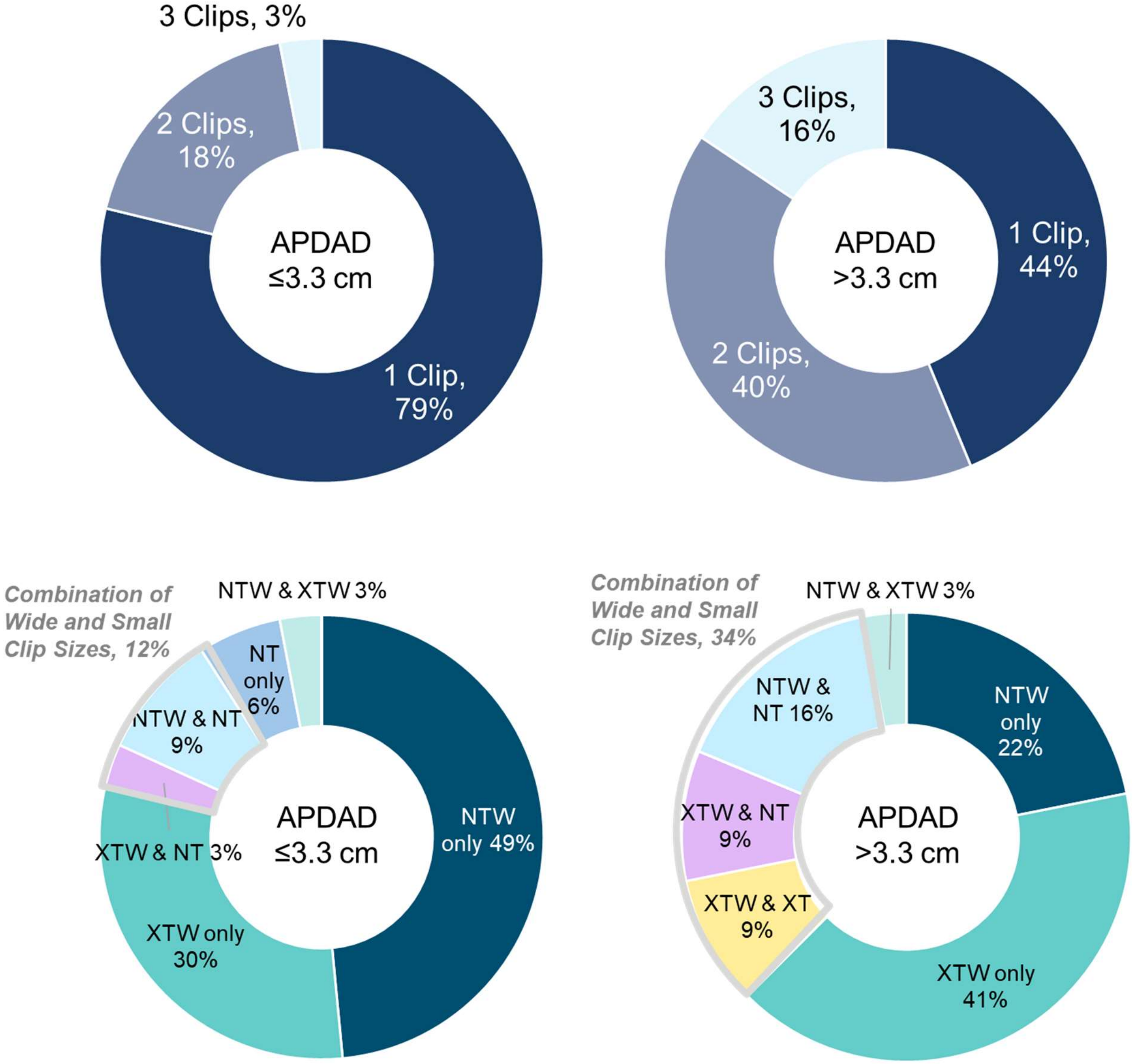


**Supplemental Figure S2. Clip Usage with the MitraClip G4 System in Atrial Secondary Mitral Regurgitation Patients by Anterior Posterior Diastolic Annular Dimension.** APDAD, anterior posterior diastolic annular dimension.

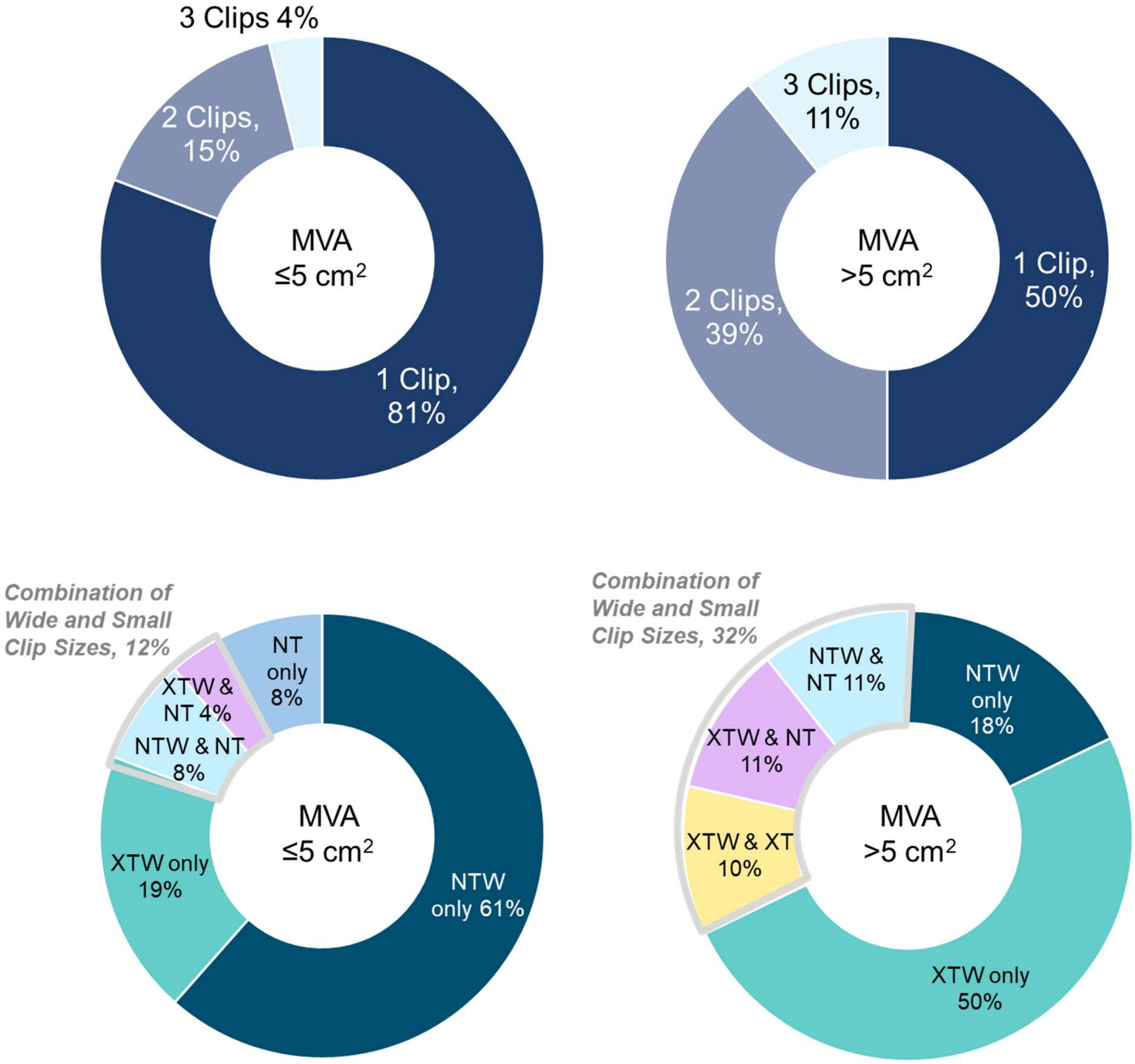


**Supplemental Figure S3. Clip Usage with the MitraClip G4 System in Atrial Secondary Mitral Regurgitation Patients by Mitral Valve Area.** MVA, mitral valve area.

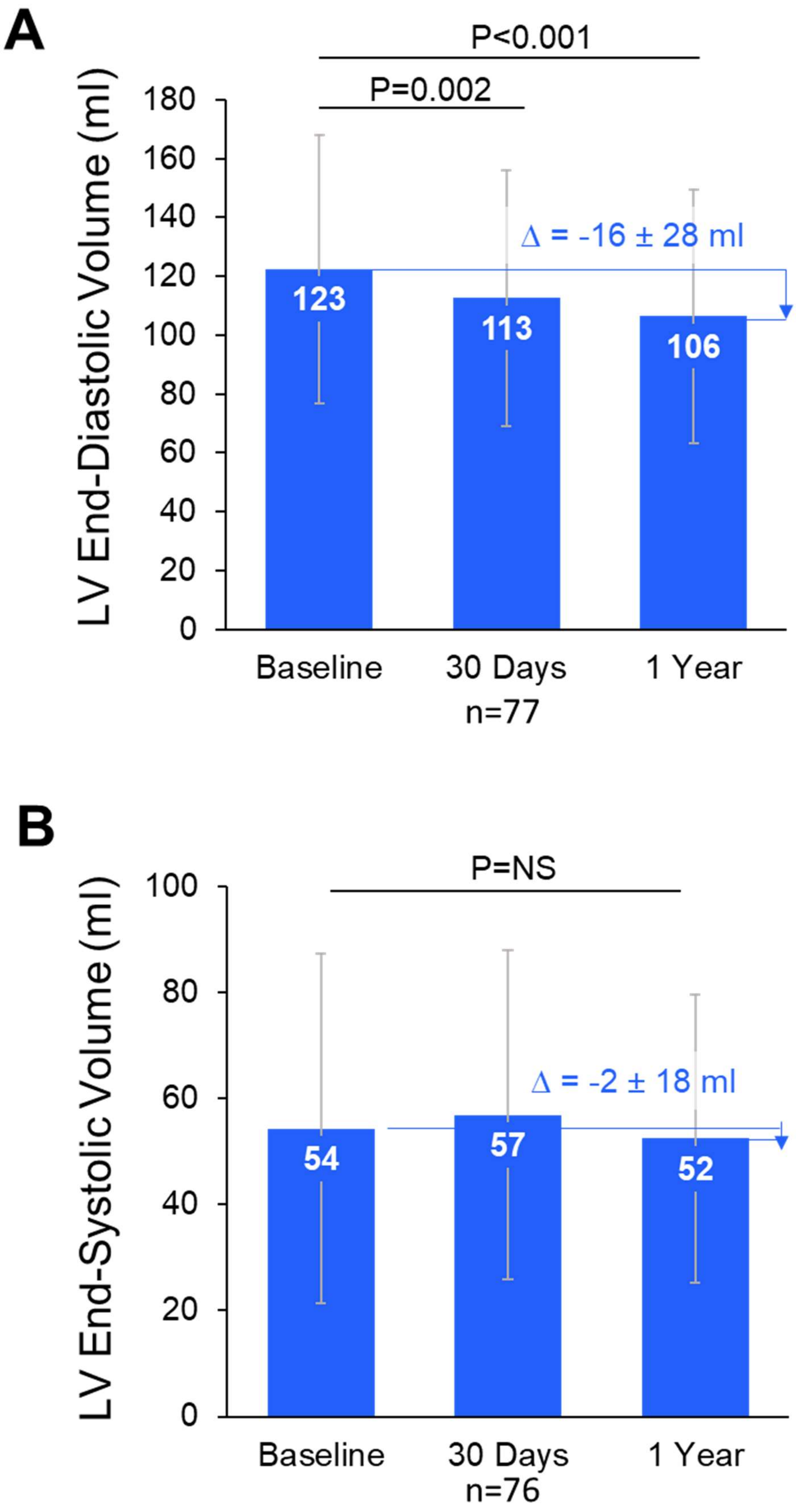
A
P<0.001
P=0.002
LV End-Diastolic Volume (ml)
180
160
140
120
100
80
60
40
20
0
Δ = -16 ± 28 ml
123
113
106
Baseline
30 Days
n=77
1 Year
B
P=NS
LV End-Systolic Volume (ml)
100
80
60
40
20
0
Δ = -2 ± 18 ml
54
57
52
Baseline
30 Days
n=76
1 Year

**Supplemental Figure S4. Left Ventricular (LV) Reverse Remodeling Through One Year**. Patients with atrial secondary mitral regurgitation showed significant decrease in LV end-diastolic volumes (A) but no change in the LV end-systolic volume (B) through one year. NS, not significant.

**Supplemental Table S1. Clip Location by Baseline Anatomic Characteristic.**

| | **A1P1**<br>**% (n/N)** | **A2P2**<br>**% (n/N)** | **A3P3**<br>**% (n/N)** |
|---|---|---|---|
| APDAD ≤3.3 | 6.5% (5/77) | 93.5% (72/77) | 11.7% (9/77) |
| APDAD >3.3 | 8.1% (6/74) | 91.9% (68/74) | 10.8% (8/74) |
| LAD ≤4.5 | 6.3% (3/48) | 89.6% (43/48) | 12.5% (6/48) |
| LAD >4.5 | 7.4% (7/94) | 92.6% (87/94) | 10.6% (10/94) |
| MVA ≤5 | 8.5% (7/82) | 90.2% (74/82) | 8.5% (7/82) |
| MVA >5 | 0.0% (0/33) | 100.0% (33/33) | 6.1% (2/33) |

APDAD, anterior posterior diastolic annular dimension; LAD, left atrial diameter; MVA, mitral valve area.

**Supplemental Table S2. Medication Usage Through 30 Days.**

| Medication | % of Patients with Atrial Secondary Mitral Regurgitation | | Change since Baseline | |
|---|---|---|---|---|
| | Baseline N=160 | 30 Days N=157 | Started Since Baseline N=157 | Stopped Since Baseline N=157 |
| ACE-Inhibitors | 32.5% (52) | 26.8% (42) | 1.9% (3) | 7.6% (12) |
| Aldosterone Antagonists | 21.3% (34) | 19.1% (30) | 7.0% (11) | 9.6% (15) |
| Angiotensin Receptor Blockers | 20.0% (32) | 19.1% (30) | 6.4% (10) | 5.7% (9) |
| Beta-Blockers | 81.3% (130) | 74.5% (117) | 3.8% (6) | 10.2% (16) |
| Diuretics | 83.1% (133) | 84.7% (133) | 8.3% (13) | 7.0% (11) |
| Novel Oral Anticoagulant | 59.4% (95) | 55.4% (87) | 6.4% (10) | 9.6% (15) |
| Antiarrhythmic | 13.8% (22) | 15.3% (24) | 5.7% (9) | 4.5% (7) |

Data presented as % (n). ACE, angiotensin-converting enzyme.
